# Robotic radical antegrade modular pancreatosplenectomy (RAMPS) versus standard retrograde pancreatosplenectomy (SRPS): study protocol for a randomized controlled trial

**DOI:** 10.1186/s13063-020-04250-0

**Published:** 2020-04-03

**Authors:** Gong Zhang, Yuhao Kang, Haifeng Zhang, Fei Wang, Rong Liu

**Affiliations:** Department of Hepatobiliary and Pancreatic Surgical Oncology, Chinese People’s Liberation Army General Hospital, 28 Fuxing Road, Beijing, 100853 China

**Keywords:** Robot-assisted, Distal pancreatectomy, Pancreatic surgery, Pancreatic cancer, RAMPS

## Abstract

**Background:**

Data from meta-analysis suggest that robotic radical antegrade modular pancreatosplenectomy (RAMPS) is a safe and effective procedure for treating adenocarcinoma in the body or tail of the pancreas, and is oncologically superior to standard retrograde pancreatosplenectomy (SRPS). RAMPS is an operation that actively expands the scope of resection, and achieves a higher R0 resection rate and lymph nodes acquisition through expanded resection. However, previous studies on RAMPS were conducted under open and laparoscopic surgery. Robotic surgery, on the other hand, plays a role in ergonomics and offers several advantages, including less fatigue, tremor filtering, 7° of wrist-like motion, motion scaling, and three-dimensional vision. At present, there is still a world-wide lack of clinical studies to observe the safety and clinical efficacy of robotic RAMPS. Hence, prospective randomized controlled trials (RCTs) comparing robotic RAMPS and SRPS are required. We begin an RCT to compare short-term surgical and oncological outcomes of robotic RAMPS and SRPS in patients undergoing distal pancreatectomy.

**Methods:**

This is a randomized, single-center clinical trial. All participants are adult patients with primary pancreatic cancer, who are undergoing RAMPS or SRPS. The primary endpoints are R0 rate (resection margins are classified by a margin to tumor distance ≥ 1 mm). The secondary endpoints are the number of harvested lymph nodes, perioperative complications and perioperative indicators (duration of surgery, blood loss, blood transfusion volume, costs).

**Discussion:**

We are undertaking a prospective RCT to evaluate the surgical and oncological outcomes of robotic RAMPS. This procedure may become a standard approach to robotic pancreatosplenectomy.

**Trial registration:**

Chinese Clinical Trial Registry: ChiCTR1900020833, Registered on 20 January 2019.

## Administrative information

Note: the numbers in curly brackets in this protocol refer to Standard protocol items: recommendation for interventional trials (SPIRIT) checklist item numbers. The order of the items has been modified to group similar items (see http://www.equator-network.org/reporting-guidelines/spirit-2013-statement-defining-standard-protocol-items-for-clinical-trials/).

**Table:** 

Title {1}	Robotic radical antegrade modular pancreatosplenectomy (RAMPS) versus standard retrograde pancreatosplenectomy (SRPS): study protocol for a randomized controlled trial
Trial registration {2a and 2b}.	Chinese Clinical Trial Registry, ID: ChiCTR1900020833. Registered on 20 January 2019
Protocol version	Protocol version is ver.2.0, 10 August 2019
Funding {4}	Department of Hepatobiliary and Pancreatic Surgical Oncology, Chinese People’s Liberation Army General Hospital (CPGH). This randomized controlled trial is conducted without external funding
Author details {5a}	All of the five protocol contributors are doctors. Rong Liu is the corresponding author. Gong Zhang is the primary investigator. Their roles are as follows: study conception and design: Rong Liu, Gong Zhang; drafting of the manuscript: Gong Zhang, YuHao Kang, Haifeng Zhang; critical revision of the manuscript: Fei Wang. All authors are from department of Hepatobiliary and Pancreatic Surgical Oncology, Chinese People’s Liberation Army General Hospital (CPGH).
Name and contact information for the trial sponsor {5b}	Name: Department of Hepatobiliary and Pancreatic Surgical Oncology, Chinese People’s Liberation Army General Hospital, Phone number: 010-66937166 Address: 28 Fuxing Road, Beijing, 100,853, China.
Role of sponsor {5c}	The sponsor played an active role in study design; collection, management, analysis, and interpretation of data; writing of the report; and the decision to submit the report for publication. It has ultimate authority on any activity in the trial.

## Introduction

### Background and rationale {6a}

Early diagnosis of pancreatic cancer is difficult, and the patient’s prognosis is extremely poor, with a 5-year survival rate of about 5% only [1]. Complete surgical resection is the only possible cure for pancreatic cancer, and microscopically margin-negative (R0) resection is the most important factor affecting postoperative survival in these patients [2–4]. Therefore, how to improve the rate of R0 resection in pancreatic surgery, and to delay and reduce local recurrence, has been a hotspot of pancreatic surgery research.

The positive rate of the peritoneal resection margin is high after SRPS, which is an important cause of tumor metastasis and recurrence. With progress in the concept of tumor treatment, surgical methods in the treatment of cancer in the pancreatic body and tail have improved. Strasberg et al. proposed RAMPS in 2003. Due to its theoretical rationality and good surgical outcome, it has attracted the attention of pancreatic surgeons. It is expected to become the standard surgical method for distal pancreatectomy [5].

The focus of RAMPS is on radical resection at the resection margin of the retroperitoneum. According to whether the tumor has invaded the posterior capsule of the pancreas, the anterior approach or the posterior approach have been used to improve the R0 resection rate of the resection margin of the retroperitoneum and the effect of radical resection of the tumor. RAMPS was reported a few years ago to significantly improve the R0 resection rate and 5-year survival rate compared with SRPS [5, 6]. However, reports in recent years have shown that despite the theoretic advantages of RAMPS over SRPS, high-level there is currently no evidence of a survival benefit with RAMPS [7, 8]. The potential advantage of RAMPS in terms of survival still needs to be proven.

The robotic surgical system plays an essential role in ergonomics and offers advantages to the surgeon, such as less fatigue, tremor filtering, 7° of wrist-like motion, motion scaling, and three-dimensional vision [9–12]. These characteristics mean that robots advantageous in delicate operations, in small spaces, in complex reconstruction, and in surgery involving blood vessels. Robotic surgery is likely to become mainstream in urological, gynecological, and other abdominal surgery in the future [13, 14].

## Objectives {7}

The aim of our study is to compare safety and the patient’s prognosis with robotic RAMPS and SRPS in the treatment of pancreatic body and tail cancer.

## Trial design {8}

This study is a single-center randomized controlled study in which patients will be assigned randomly into control and trial groups.

## Methods: participants, interventions and outcomes

### Study setting {9}

**Fig. 1:**
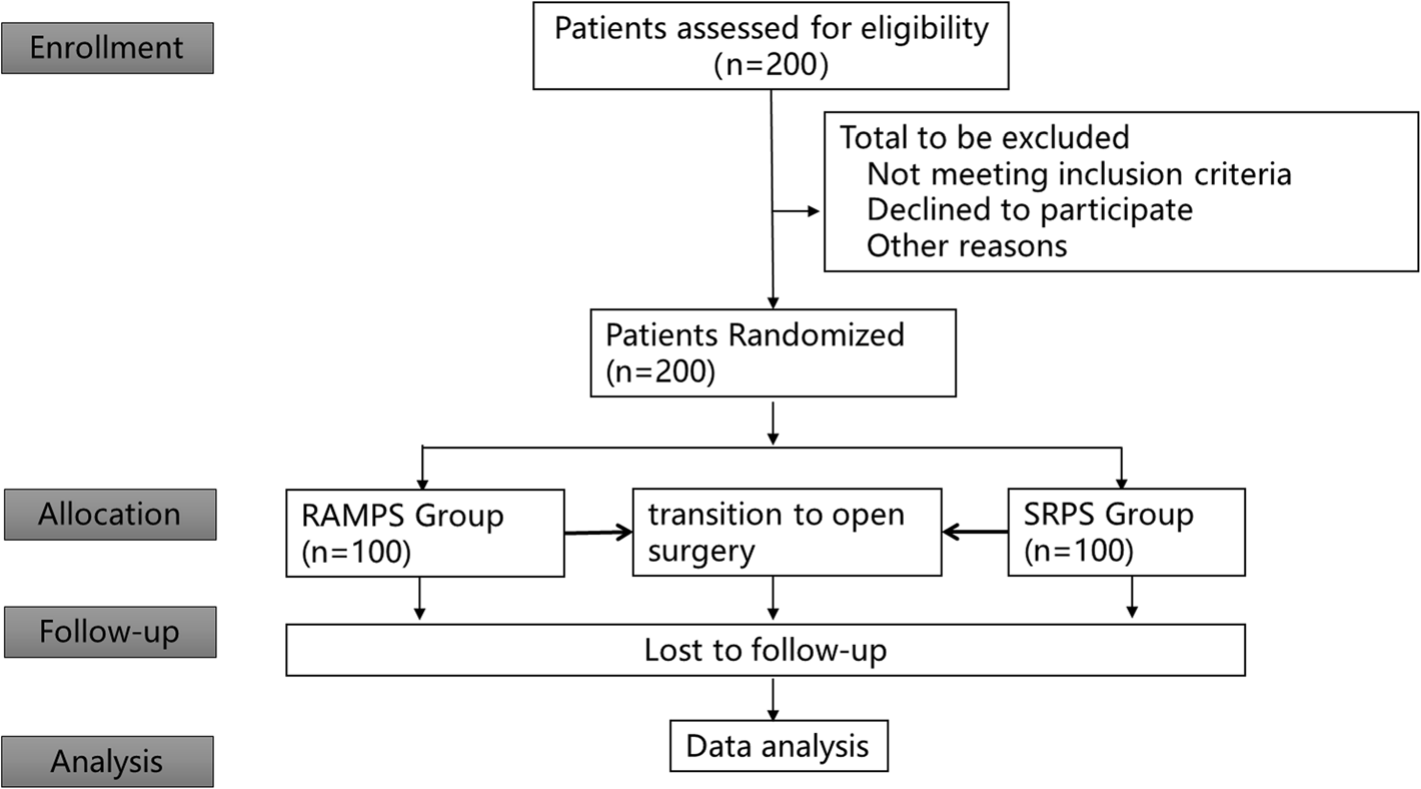
Consolidated standards of reporting trials (CONSORT) flow diagram. RAMPS, radical antegrade modular pancreatosplenectomy; SRPS, standard retrograde pancreatosplenectomy

From August 2019 to 31 September 2022, patients will be selected from the Chinese People’s Liberation Army General Hospital (CPGH) for treatment. All patients must have been diagnosed with pancreatic body and tail cancer and meet the inclusion criteria. A total of 256 patients are scheduled to be included in the study. After confirmation of the eligibility criteria, including written informed consent, registration is made in the central registry in CPGH. Each patient will be randomly assigned a number in the central registry. Then, patients are randomized in a 1:1 allocation ratio either to arm A (RAMPS) or to arm B (SRPS), with a random block size (Fig. 1).

### Eligibility criteria {10}

#### Inclusion criteria

The inclusion criteria are:

1. Informed consent signed by the patient or his or her legal agent;
2. Compliance with the study plan and follow-up procedure;
3. Men or women ages 18–70 years;
4. No surgical contraindications, able to tolerate radical surgery, Eastern Cooperative Oncology Group (ECOG) behavioral status score 0–1, life expectancy ≥ 12 weeks, American Society of Anestheologists (ASA) score ≤ 2;
5. Tumor diagnosed as resectable on preoperative pathological examination or clinical judgment;
6. Tumor meets the indications required for resection of pancreatic somatococcygeal carcinoma by robot.

#### Exclusion criteria

The exclusion criteria are:

1. Malignant tumors elsewhere in the body within the last 5 years;
2. Brain, lung, bone, or abdominal lymph node metastases;
3. Severe cardiopulmonary function, or infection affecting liver or renal function;
4. Pregnancy or lactation.

#### Participating surgeons

Differences in the surgical experience of surgeons may lead to differences in the incidence of complications. By analyzing the perioperative data from the first 100 cases of robotic pancreaticoduodenectomy performed by a single surgeon in our center, we found that the surgeon's learning cure was completed after performing 40 such operations. The duration of surgery, intraoperative blood loss and incidence of complications in the patients were significantly decreased [15]. In this research, our surgical team consists of three surgeons and each of these clinicians meet the participation requirements and have received adequate training prior to participating in the study. Patients will be randomly assigned to each surgeon’s group.

### Who will take informed consent? {26a}

The surgeon, Dr Liu, will recruit the patient when discussing the pancreatosplenectomy treatment with the patient. Patients from CPGH will be selected for treatment from August 2019 to 31 September 2022. The trial was consistent with the principles of the Declaration of Helsinki and in accordance with the Medical Research Involving Human Subjects Act (WMO), and has been approved by the medical ethics committee of CPGH. Data are processed anonymously to protect the participants’ privacy. Written informed consent for the study will be obtained from each patient before surgery.

### Additional consent provisions for collection and use of participant data and biological specimens {26b}

On the consent form, if the participant opts out of the trial, the participant is asked if he or she agrees that their data may be used. Participants will also be required to allow the research team to share relevant data with people from the university or relevant authorities.

## Interventions

### Explanation for the choice of comparators {6b}

Previous studies on RAMPS were conducted under laparotomy and laparoscopy. There is still a world-wide lack of clinical studies on the safety and clinical efficacy of robotic RAMPS. We intend to conduct an RCT to compare short-term surgical oncological outcomes of robotic RAMPS and robotic SRPS in patients undergoing distal pancreatectomy. Through this study we want to find out whether RAMPS performs better than SRPS in robotic surgery.

### Intervention description {11a}

#### Surgical technique

##### Robotic radical antegrade modular pancreatosplenectomy

**Fig. 2:**
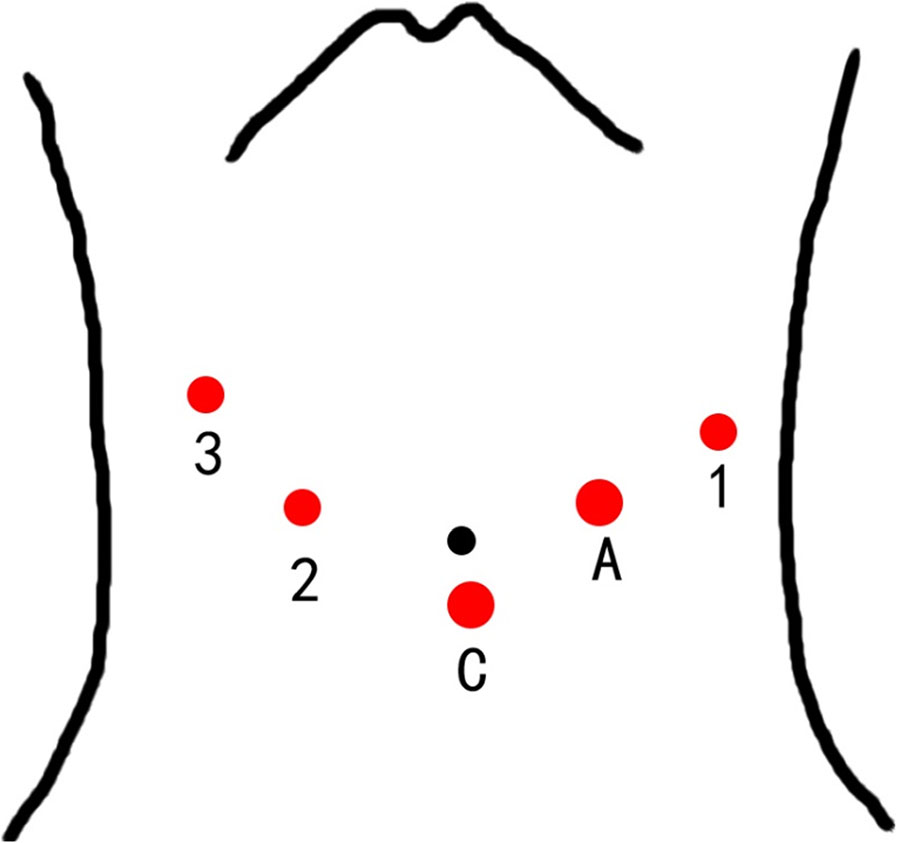
Robotic port placement for robotic distal pancreatectomy (RDP). C, 12-mm trocar for camera; A, 12-mm trocar for assistant instruments; 1, 8-mm trocar for right robotic arm; 2, 8-mm trocar for left robotic arm; 3, 8-mm trocar for the fourth robotic arm

All robotic distal pancreatectomy (RDP) procedures will be performed using the da Vinci™ Si Surgical System (Intuitive, Sunnyvale, CA, USA). Once general anesthesia has taken effect, the patient is placed in a supine position. Our surgical procedures of RDPS and LDPS have been described previously [16, 17]. The layout of the trocar is shown in Fig. 2 [17]. Diagnostic laparoscopy is performed to rule out metastasis. The gastric colon ligament is opened and the superior mesenteric vein is separated at the lower margin of the pancreatic neck. The little omentum capsule is then opened to dissect the common hepatic artery, and para-hepatic arterial lymph nodes are dissected (groups 8a and 8p). The gastroduodenal artery is isolated, and then the superior portal vein of the pancreas is exposed. The pancreatic neck tunnel is located and the pancreatic neck is disconnected. Lymph nodes around the celiac trunk (nine groups) and fibrous adipose tissue are dissected. The splenic artery is isolated along the celiac trunk, is ligated and severed at the root, and the left gastric artery is severed when necessary. The distal pancreas is pulled from right to left, the splenic vein is severed from the root, and the proximal end is closed with 5-0 prolene continuous suture. Lymph nodes are dissected downward from the celiac trunk and the periceliac nerve plexus to the superior mesenteric artery, and the left lymph node of the superior mesenteric artery is dissected (group 14c and group 14d). Dissection is continued posteriorly to reveal the leading edge of the left renal vein and left adrenal vein. The specimen is redirected to the left to ensure that the anatomical resection plane is located behind Gerota’s fascia. The anterior approach should be close to the left renal vein, renal capsule, and the front edge of the left adrenal surface to clear the retroperitoneal tissue (this is the anterior RAMPS). The posterior approach requires resection of the left adrenal gland and its surrounding tissue (posterior RAMPS). At the same time, the upper and lower edges of the pancreas are freed, and then the ligaments around the spleen are removed, and the specimen is finally removed.

##### Robotic standard retrograde pancreatosplenectomy

Anesthesia and positioning of the trocar are as described above for RAMPS. The colonic ligaments are opened and the spleen-stomach ligaments, splenic-colonic ligaments, and splenic-diaphragmatic ligaments are separated to free the spleen. The pancreas is separated from the retroperitoneum from left to right and is disconnected about 2 cm away from the distal end of the tumor. Then the pancreatic stump is sutured to stop the bleeding.

#### Intra-abdominal drainage

A drainage tube will be placed to assess the pancreatic fistula as the primary endpoint before the abdomen is closed. The number and location of the inserted drainage tubes will be recorded in the data sheet, and we will specify when to remove the drain.

### Criteria for discontinuing or modifying allocated interventions {11b}

#### The rejection and withdrawal criteria

The criteria for rejection and withdrawal are (1) the patient does not meet the inclusion criteria of the study; (2) the clinical data obtained after inclusion are incomplete, and further clinical statistical analysis cannot be conducted; (3) the patients experienced serious adverse events/reactions related to the treatment regimen, and the investigator considered it necessary to withdraw them from the trial; (4) during the trial, the patient’s condition continued to deteriorate and dangerous events might occur, so the researcher considered it necessary to withdraw the patient from the clinical trial; (5) patients who voluntarily withdrew during the trial - all patients who gave informed consent and were eligible to participate in the trial at screening, regardless of when or where they withdrew, were classified as dropouts: they did not complete the observation period specified in the agreement; and (6) poor treatment compliance affecting the determination of efficacy and safety.

### Strategies to improve adherence to interventions {11c}

#### Concurrent and supportive treatments

Adherence is not an issue for patients as this is a surgical trial. We will determine details of the operation postoperatively by checking the surgical record.

### Relevant concomitant care permitted or prohibited during the trial {11d}

The surgeon will determine the use of antibiotics, blood products, analgesics, H2 blockers, and proton pump inhibitors based on perioperative management. Octreotide will be routinely applied to prevent pancreatic fistula. Abdominal ultrasonography will be performed regularly to determine the presence of peritoneal effusion. The serum amylase and bilirubin will be measured regularly in the drainage fluid of the abdominal drainage tube after operation. Implementing robotic RAMPS or robotic SRPS will not require alteration to usual care pathways and these will continue in both trial arms.

### Provisions for post-trial care {30}

There is no anticipated harm or need for compensation due to trial participation.

### Outcomes {12}

#### Endpoints

The primary efficacy endpoint of the trial will be the rates of resection margins. In the case of malignant pancreatic disease, resection margins are classified by a margin to tumor distance ≥ 1 mm (R0), a margin to tumor distance < 1 mm (R1) or a macroscopically positive margin (R2) [18]. The secondary endpoints are (1) the number of harvested lymph nodes; (2) retrieval postoperative complications including pancreatic fistula, intra-abdominal abscess, and anastomotic leakage, according to the Clavien-Dindo classification (Table 1): complications classified as higher than grade II are regarded as clinically significant [19]; and (3) perioperative indicators; such as duration of the operation, blood loss, blood transfusion volume, rate of transition to open surgery, postoperative hospitalization days, and hospital costs.

**Table 1:** Complication grades according to the Clavien-Dindo classication scheme

Grade	Definition
V	Death of patient
IV	Life-threatening complication. Requiring intensive care unit management
IVa	Single organ dysfunction
IVb	Multi-organ dysfunction
III	Requiring surgical, endoscopic, or radiological intervention
IIIa	Intervention not under general anesthesia
IIIb	Intervention under general anesthesia
II	Requiring pharmacological treatment with drugs other than those allowed for grade I complications
I	Any deviation from the normal postoperative course without the need for pharmacological treatment or surgical, endoscopic, and radiological intervention

### Participant timeline {13}

An independent research physician will not be involved in the treatment and monitoring of patients in the surgical room and enter all necessary data into the prepared clinical report form (CRF). The CRF will be completed as soon as possible, preferably on the day of the patient’s visit and treatment (Table 2). Reasonable explanations should be given for all missing data. The complete CRF page will be examined for completeness and reasonableness by the principal investigator (PI) and responsible supervisors.

**Table 2:** Flow chart of the trial

	Screening					
	Visit 1 Before surgery	Visit 2 Day of surgery	Visit 3 POD 1	Visit 4 POD 3	Visit 5 POD 7	Visit 5 POD 30
Informed consent	x					
Personal data	x					
Physical examination	x					
Previous medical history	x					
Inclusion/exclusion criteria	x					
CT/MRI	x					
Blood tests	x		x	x	x	x
Trial intervention		x				
Intraoperative outcomes		x				
Complications		x				
Adverse events		x	x	x	x	x
Postoperative outcomes			x	x	x	x

*CT* computed tomography, *MRI* magnetic resonance imaging, *POD* postoperative day

### Sample size {14}

Determination of the marginal resection rate is the main endpoint of this study. Published reports describe an R0 resection rate of 50–74% in distal adenocarcinoma in studies with large sample sizes (*n* > 100 patients) [20, 21]. A systematic review of radical antegrade modular pancreatosplenectomy, which identified 13 observational studies involving 354 patients undergoing RAMPS, showed that the R0 resection rate was 88% [22]. According to these studies, we used a two-sided log-rank test, which requires about 23–123 patients in each group with 80% power at the 0.05 level of significance (NCSS and PASS 11 (NCSS Statistical Software, Kaysville, UT, USA)). Considering that there is a large gap in the estimated quantity of samples, we plan to collect 90 samples in each group. Considering a dropout rate of 10%, the total sample size required is 200 patients. After 50 samples have been collected in each group, we will make a mid-term comparison to determine whether there are significant differences between the groups. Based on the results, we decide whether or not to conduct further data collection.

### Recruitment {15}

Because we are a top hospital in China, we believe we can collect enough patient data within the planned timescale.

## Assignment of interventions: allocation

### Sequence generation {16a}

Each patient will be assigned a computer-generated random number in the central registry.

### Concealment mechanism {16b}

Patients are randomized in a 1:1 allocation ratio either to arm A (RAMPS) or to arm B (SRPS), with a random block size (Fig. 1).

### Implementation {16c}

The central registry in CPGH will generate the allocation sequence, enroll participants and assign participants to interventions.

## Assignment of interventions: blinding

### Who will be blinded {17a}

The experiment was a single-blind trial. Patients will not know their grouping. Surgeons perform operations according to their group.

### Procedure for unblinding if needed {17b}

After the patient is discharged from hospital, we will send the patient’s surgical procedure in the form of medical records.

## Data collection and management

### Plans for assessment and collection of outcomes {18a}

The data manager will use two input methods to enter data from the CRF table into the ResMan database. The inspector will examine each item in the database, report inconsistent result values, validate each item in the original questionnaire, and make corrections as necessary.

### Plans to promote participant retention and complete follow up {18b}

Not applicable.

### Data management {19}

The CPGH will be responsible for data management and statistical analysis in the study. The corresponding tables of codes and informed consent forms will be kept strictly secure in the CPGH file library. All required parameters will be collected in SPSS data files (SPSS version 25, IBM statistics, Chicago, IL, USA).

### Confidentiality {27}

Participants’ medical records will be kept at the hospital. Researchers, research institutions and ethics committees will be allowed access to the records. The study will not reveal the individual identities of the participants. Participants can request access to their personal information (such as address and contact information) at any time and can modify this information if necessary.

### Plans for collection, laboratory evaluation, and storage of biological specimens for genetic or molecular analysis in this trial or for future use {33}

In this trial we will collect blood and drainage fluid samples from patients for laboratory testing.

## Statistical methods

### Statistical methods for primary and secondary outcomes {20a}

Statistical analysts do not participate in clinical observation. They will be blinded to allocation and responsible for statistical analysis of research data and timely delivery of statistical reports to the research director. All analyses will be performed and reported in accordance with the Consolidated standards of reporting trials (CONSORT) statement and the ICH E9 “Statistical Principles in Clinical Trials”. Primary and secondary outcomes will be cross-checked against data. The measurement data are expressed as mean plus/minus standard deviation. The normality test and homogeneity test of variance will be performed first. The independent sample *t* test will be used to compare normally distributed continuous variables, and the values will be represented as the mean with standard deviation. Continuous non-normally distributed variables will be compared using the Mann-Whitney *U* test, and the values will be expressed as the median of the quartile spacing. The categorical variables are compared using the chi-square test or the Fisher’s exact test, and values will be expressed as proportions with corresponding risk ratios and 95% confidence intervals. *P* < 0.05 indicates statistical significance. Statistical analysis will be performed using SPSS 20.0 software.

### Interim analyses {21b}

Statistical analysis will be performed when the total number of samples collected reaches 100. The primary investigator will obtain these interim results and decide whether to continue the experiment. We will discontinue the trial if the safety of the RAMPS surgery group is much lower than that of the control group in the outcome of the interim data.

### Methods for additional analyses (e.g. subgroup analyses) {20b}

We plan to do subgroup analysis by gender or surgeon undertaking the operations in the future.

### Methods in analysis to handle protocol non-adherence and any statistical methods to handle missing data {20c}

We will exclude patients who do not receive the intervention and whose primary data are missing.

### Plans to give access to the full protocol, participant-level-data and statistical code {31c}

The full protocol is available on request from the corresponding author.

## Oversight and monitoring

### Composition of the coordinating center and trial steering committee {5d}

The data monitoring committee (DMC) consists of principals, data managers, data monitors, and statistical analysts. It is independent from the sponsor and competing interests

### Composition of the data monitoring committee, its role and reporting structure {21a}

During the study, the DMC will be established to conduct periodic interim evaluations and, where appropriate, to optimize the study based on the results of the interim evaluations. When there are obvious differences such as in the safety gap between the two groups of experiments, the DMC is authorized to discontinue the trial.

### Adverse event reporting and harms {22}

Any adverse medical events that occur in patients during the observational clinical study are considered adverse events (AE). Complications resulting from surgery, such as pancreatic fistula, postoperative bleeding, and death, are considered serious AE and are reported to the medical supervisor. AE report forms will be filled out during the trial period. We will record the timing, severity, and duration of AE, the actions taken, and the outcome of the AE.

### Frequency and plans for auditing trial conduct {23}

During the implementation of the project, the DMC will conduct regular or irregular review and random inspection of the original test data and check the compliance of the study.

### Plans for communicating important protocol amendments to relevant parties (e.g. trial participants, ethical committees) {25}

When major changes occur in the study process, we will first notify the sponsor, then the principal investigator (PI) will notify the centers and that a copy of the revised protocol will be sent to the PI to add to the investigator site file. Any deviations from the protocol will be fully documented using a breach report form. Then we will update the protocol in the clinical trial registry.

## Dissemination plans {31a}

We will publish the results in the journal after all data have been collected and counted.

## Discussion

Trendelemburg completed the world’s first resection of the tail of the pancreas in 1882 [23]. Mayo standardized the procedure in 1913. In this way, the spleen was gradually removed from left to right, and the pancreas was gradually severed to remove the lesion [24]. But this type of surgery has disadvantages. During the process of resection, the tumor may be squeezed, causing metastasis or recurrence of the tumor.

In 2003, Strasberg modified the traditional method of pancreatic tail resection. The pancreatic neck was first separated and the superior mesenteric arteries and veins, splenic vessels, and celiac trunk were exposed. The corresponding lymph nodes were then dissected and the splenic vessels were severed. The tumor and spleen were finally resected from right to left. This operation mode can be in line with lymph node drainage mode to clean lymph nodes, and can achieve “no-touch” in the tumor resection, so as to improve the rate of tumor resection of R0 [5].

There have been some studies comparing the advantages and disadvantages of SRPS and RAMPS, suggesting that RAMPS is safe and feasible [25] and has a better R0 resection rate than traditional tail pancreatectomy (70–80%) [26]. In the system evaluation of Zhou in 2017, 13 clinical studies including 354 patients with RAMPS were included, and the R0 resection rate reached 88%, and the 5-year survival rate reached 37%. Compared with SRPS, RAMPS is associated with less bleeding, more lymph node dissection, and a higher R0 resection rate [22].

But there has been a lack of studies comparing the two approaches to robotic surgery. To evaluate the surgical and oncological outcomes of robotic RAMPS, we therefore will undertake a prospective RCT. This procedure may become a standard approach to robotic pancreatosplenectomy. In this way, the most beneficial technique can be selected for individual patients.

## Trial status

We are currently recruiting participants. The latest version is version 2.0 on 10 August 2019. The first participant was recruited on 6 September 2019, and the recruitment is expected to be completed in December 2020.

## Data Availability

Data sets used or analyzed in the current study may be provided upon reasonable request of the corresponding author.

## Abbreviations

AE: Adverse events
CONSORT: Consolidated standards of reporting trials
CPGH: Chinese People’s Liberation Army General Hospital
CRF: Clinical report form
OS: Overall survival
PI: Principal investigator
RAMPS: Radical antegrade modular pancreatosplenectomy
RCT: Randomized controlled trial
RDP: Robotic distal pancreatectomy
RFS: Relapse-free survival
SRPS: Standard retrograde pancreatosplenectomy

## References

[1] Hidalgo M (2010). Pancreatic cancer. N Engl J Med.

[2] Hartwig W, Hackert T, Hinz U, Gluth A, Bergmann F, Strobel O, Buchler MW, Werner J (2011). Pancreatic cancer surgery in the new millennium: better prediction of outcome. Ann Surg.

[3] Marzano E, Piardi T, Soler L, Diana M, Mutter D, Marescaux J, Pessaux P (2013). Augmented reality-guided artery-first pancreatico-duodenectomy. J Gastrointest Surg.

[4] Yamamoto T, Yagi S, Kinoshita H, Sakamoto Y, Okada K, Uryuhara K, Morimoto T, Kaihara S, Hosotani R (2015). Long-term survival after resection of pancreatic cancer: a single-center retrospective analysis. World J Gastroenterol.

[5] Strasberg SM, Drebin JA, Linehan D (2003). Radical antegrade modular pancreatosplenectomy. Surgery.

[6] Strasberg SM, Linehan DC, Hawkins WG (2007). Radical antegrade modular pancreatosplenectomy procedure for adenocarcinoma of the body and tail of the pancreas: ability to obtain negative tangential margins. J Am Coll Surg.

[7] Chun YS (2018). Role of radical antegrade modular pancreatosplenectomy (RAMPS) and pancreatic cancer. Ann Surg Oncol.

[8] Zhou Q, Fengwei G, Gong J, Xie Q, Liu Y, Wang Q, Lei Z (2019). Assessement of postoperative long-term survival quality and complications associated with radical antegrade modular pancreatosplenectomy and distal pancreatectomy: a meta-analysis and systematic review. BMC Surg.

[9] Orady M, Hrynewych A, Nawfal AK, Wegienka G (2012). Comparison of robotic-assisted hysterectomy to other minimally invasive approaches. JSLS.

[10] Sohn W, Lee HJ, Ahlering TE (2013). Robotic surgery: review of prostate and bladder cancer. Cancer J.

[11] Zureikat AH, Moser AJ, Boone BA, Bartlett DL, Zenati M, Zeh HJ (2013). 250 robotic pancreatic resections: safety and feasibility. Ann Surg.

[12] Memeo R, Sangiuolo F, de Blasi V, Tzedakis S, Mutter D, Marescaux J, Pessaux P (2016). Robotic pancreaticoduodenectomy and distal pancreatectomy: state of the art. J Visc Surg.

[13] Khosla A, Wagner AA (2016). Robotic surgery of the kidney, bladder, and prostate. Surg Clin North Am.

[14] Kornaropoulos M, Moris D, Beal EW, Makris MC, Mitrousias A, Petrou A, Felekouras E, Michalinos A, Vailas M, Schizas D (2017). Total robotic pancreaticoduodenectomy: a systematic review of the literature. Surg Endosc.

[15] Zhang T, Zhao ZM, Gao YX, Lau WY, Liu R (2019). The learning curve for a surgeon in robot-assisted laparoscopic pancreaticoduodenectomy: a retrospective study in a high-volume pancreatic center. Surg Endosc.

[16] Liu R, Zhang T, Zhao Z, Tan X, Zhao G, Zhang X, Xu Y (2017). The surgical outcomes of robot-assisted laparoscopic pancreaticoduodenectomy versus laparoscopic pancreaticoduodenectomy for periampullary neoplasms: a comparative study of a single center. Surg Endosc.

[17] Liu R, Liu Q, Zhao ZM, Tan XL, Gao YX, Zhao GD (2017). Robotic versus laparoscopic distal pancreatectomy: a propensity score-matched study. J Surg Oncol.

[18] Buchler MW, Werner J, Weitz J (2010). R0 in pancreatic cancer surgery: surgery, pathology, biology, or definition matters?. Ann Surg.

[19] Dindo D, Demartines N, Clavien PA (2004). Classification of surgical complications: a new proposal with evaluation in a cohort of 6336 patients and results of a survey. Ann Surg.

[20] Kooby DA, Hawkins WG, Schmidt CM, Weber SM, Bentrem DJ, Gillespie TW, Sellers JB, Merchant NB, Scoggins CR, Martin RC (2010). A multicenter analysis of distal pancreatectomy for adenocarcinoma: is laparoscopic resection appropriate?. J Am Coll Surg.

[21] de Rooij T, Tol JA, van Eijck CH, Boerma D, Bonsing BA, Bosscha K, van Dam RM, Dijkgraaf MG, Gerhards MF, van Goor H (2016). Outcomes of distal pancreatectomy for pancreatic ductal adenocarcinoma in the Netherlands: a nationwide retrospective analysis. Ann Surg Oncol.

[22] Zhou Y, Shi B, Wu L, Si X (2017). A systematic review of radical antegrade modular pancreatosplenectomy for adenocarcinoma of the body and tail of the pancreas. HPB (Oxford).

[23] Sulkowski U, Meyer J, Reers B, Pinger P, Waldner M (1991). The historical development of resection surgery in pancreatic carcinoma. Zentralbl Chir.

[24] Mayo WJ (1913). I. The surgery of the pancreas: I. Injuries to the pancreas in the course of operations on the stomach. II. Injuries to the pancreas in the course of operations on the spleen. III. Resection of half the pancreas for tumor. Ann Surg.

[25] Grossman JG, Fields RC, Hawkins WG, Strasberg SM (2016). Single institution results of radical antegrade modular pancreatosplenectomy for adenocarcinoma of the body and tail of pancreas in 78 patients. J Hepatobiliary Pancreat Sci.

[26] Lillemoe KD, Kaushal S, Cameron JL, Sohn TA, Pitt HA, Yeo CJ (1999). Distal pancreatectomy: indications and outcomes in 235 patients. Ann Surg.

